# Lytic polysaccharide monooxygenases (LPMOs) facilitate cellulose nanofibrils production

**DOI:** 10.1186/s13068-019-1501-0

**Published:** 2019-06-24

**Authors:** Céline Moreau, Sandra Tapin-Lingua, Sacha Grisel, Isabelle Gimbert, Sophie Le Gall, Valérie Meyer, Michel Petit-Conil, Jean-Guy Berrin, Bernard Cathala, Ana Villares

**Affiliations:** 1 UR1268 Biopolymères Interactions Assemblages, INRA, 44316 Nantes, France; 2 InTechFibres Division, FCBA, Domaine Universitaire, CS 90252, 39044 Grenoble Cedex 9, France; 3 Biodiversité et Biotechnologie Fongiques, INRA, Aix Marseille University, UMR1163, 13009 Marseille, France; 4 CTP, Domaine Universitaire, CS 90252, 39044 Grenoble Cedex 9, France

**Keywords:** Lytic polysaccharide monooxygenase (LPMO), Biorefinery, Nanofibrillated cellulose (NFC), Microfluidizer, Molar mass, 13C CP/MAS NMR

## Abstract

**Background:**

Lytic polysaccharide monooxygenases (LPMOs) are copper-dependent enzymes that cleave polysaccharides through an oxidative mechanism. These enzymes are major contributors to the recycling of carbon in nature and are currently used in the biorefinery industry. LPMOs are commonly used in synergy with cellulases to enhance biomass deconstruction. However, there are few examples of the use of monocomponent LPMOs as a tool for cellulose fibrillation. In this work, we took advantage of the LPMO action to facilitate disruption of wood cellulose fibers as a strategy to produce nanofibrillated cellulose (NFC).

**Results:**

The fungal LPMO from AA9 family (*Pa*LPMO9E) was used in this study as it displays high specificity toward cellulose and its recombinant production in bioreactor is easily upscalable. The treatment of birchwood fibers with *Pa*LPMO9E resulted in the release of a mixture of C1-oxidized oligosaccharides without any apparent modification in fiber morphology and dimensions. The subsequent mechanical shearing disintegrated the LPMO-pretreated samples yielding nanoscale cellulose elements. Their gel-like aspect and nanometric dimensions demonstrated that LPMOs disrupt the cellulose structure and facilitate the production of NFC.

**Conclusions:**

This study demonstrates the potential use of LPMOs as a pretreatment in the NFC production process. LPMOs weaken fiber cohesion and facilitate fiber disruption while maintaining the crystallinity of cellulose.

**Electronic supplementary material:**

The online version of this article (10.1186/s13068-019-1501-0) contains supplementary material, which is available to authorized users.

## Background

Replacement of the fossil oil-based products with renewable materials is a critical demand to implement bioeconomy. Cellulose appears as a potential candidate since it is the most abundant renewable polymer produced on Earth through photosynthesis [1, 2]. Since a few decades, interest for cellulose-based materials has tremendously increased by the renewal of nanocelluloses [3–8]. The term nanocellulose is employed when the cellulosic objects present at least one dimension in the nanoscale (1–100 nm). Nanocelluloses are usually divided into two main types: cellulose nanocrystals (CNCs) and nanofibrillated cellulose (NFC). CNCs have been reported for the first time in the 1950s and are commonly obtained by acid hydrolysis resulting in nanorods with high aspect ratio derived from the crystalline part of the fibers [9, 10]. NFC is obtained by mechanical delamination of the fibers as firstly reported in the early 1980s. It consists in flexible fibrils with length higher than CNCs and lateral dimensions depending on the production process but ranging from few nanometers to tens of nanometers [11, 12]. Both CNC and NFC are used in many applications thanks to their amazing properties such as high mechanical strength, ability to stabilize emulsions, gas barrier, dispersing properties and others [6, 7, 13–17]. Methods of NFC production from wood cellulosic fibers are numerous but most of them can be summarized in two main steps [8]. The first one consists in a pretreatment that can be either chemical (by 2,2,6,6-tetramethylpiperidine-1-oxyl (TEMPO)-mediated oxidation or carboxymethylation) or enzymatic (endoglucanase and/or xylanase treatment) [18–25]. Mechanical nanofibrillation is achieved in the second step by different technologies such as high-pressure homogenization, microfluidization or grinding, among the most widely reported [6, 8]. The pretreatment step is mandatory since starting fibers cannot be otherwise processed. The goal of the pretreatment step is to (i) weaken the fiber cohesion to allow fiber processing, (ii) lower the energy consumption of the mechanical process and (iii) improve the final quality of NFC. Thus, the efficiency of the pretreatment step is a key point in the process since it greatly impacts the quality of the final NFC and also the energy efficiency of the process. Accordingly, the development of innovative pretreatments for NFC production is still an intense field of investigation.

Lytic polysaccharide monooxygenases (LPMOs) are copper-dependent enzymes that cleave polysaccharides through an oxidative mechanism. They are classified as Auxiliary Activity (AA) enzymes by the Carbohydrate-Active enZymes database (CAZy; http://www.cazy.org). The proposed mechanism of action consists of the cleavage of cellulose by the insertion of oxygen at C1 and/or C4, with the subsequent formation of a lactone, which is spontaneously hydrolyzed to aldonic acid or a ketoaldose, respectively [26]. These enzymes have been initially used in synergy with glycoside hydrolases to boost the saccharification of plant biomass, and they have been incorporated in last-generation commercial enzyme cocktails for the production of biofuels [27]. Recent studies have shown that LPMOs may be used in synergy with cellulases and/or xylanases to facilitate the deconstruction of cellulose fibers in an attempt to produce NFC [28, 29]. In that line of research, we had previously demonstrated that monocomponent LPMOs can disrupt the cellulose fibers structure by the creation of nicking points that weaken the fiber cohesion [30]. In this study, we assessed the use of LPMOs as pretreatment of cellulosic fibers for NFC production. Bleached birchwood Kraft pulp was submitted to LPMO action and then processed with a microfluidizer to demonstrate the industrial relevance of the process. LPMO-treated fibers were successfully dispersed at nanoscale while untreated fibers could not be processed. The structure of the NFC all along the dispersion treatment was investigated by using MorFi analysis, optical and atomic force microscopy and solid-state NMR. Monosaccharide composition of the fibers and molar mass distribution of cellulose chains were also determined. Results provide new insights on the LPMO action as well as the proof of concept that LPMOs can be efficiently implemented for NFC production.

## Results

### Production of *Pa*LPMO9E in bioreactor

An important feature to consider upon production of recombinant LPMOs is the perfect processing of the signal peptide during secretion to ensure correct binding of the catalytic copper ion by the histidine brace that includes the N-terminal histidine residue. Optimal processing of signal peptides during heterologous production is protein dependent, and heterogeneity on N-terminal sequences is a recurrent problem [31]. In *P. pastoris*, the use of the α-mating factor (α-MF) as signal peptide is sometimes associated with incorrect cleavage by the Ste13 protease [31]. Therefore, we designed a plasmid construct using the native signal sequence of the *Pa*LPMO9E encoding gene to foster recombinant protein production in *P. pastoris*. Using this strategy, the production yield of *Pa*LPMO9E, assessed by measuring the recombinant protein production in the medium after induction, significantly increased (about twofold) when the native signal sequence was used. Beyond the fact that the recombinant LPMO was expressed to high level, N-terminal sequencing indicated that the processing of the signal peptide was fully achieved yielding a functional enzyme batch.

Taking advantage of this new construct, we set up the recombinant production of *Pa*LPMO9E in bioreactor up to 2 g of protein per liter of culture after 5 days (Additional file 1: Fig. S1) enabling the enzymatic processing of wood cellulosic fibers under industrially relevant conditions.

### LPMO pretreatment allows the production of NFC

**Fig. 1:**
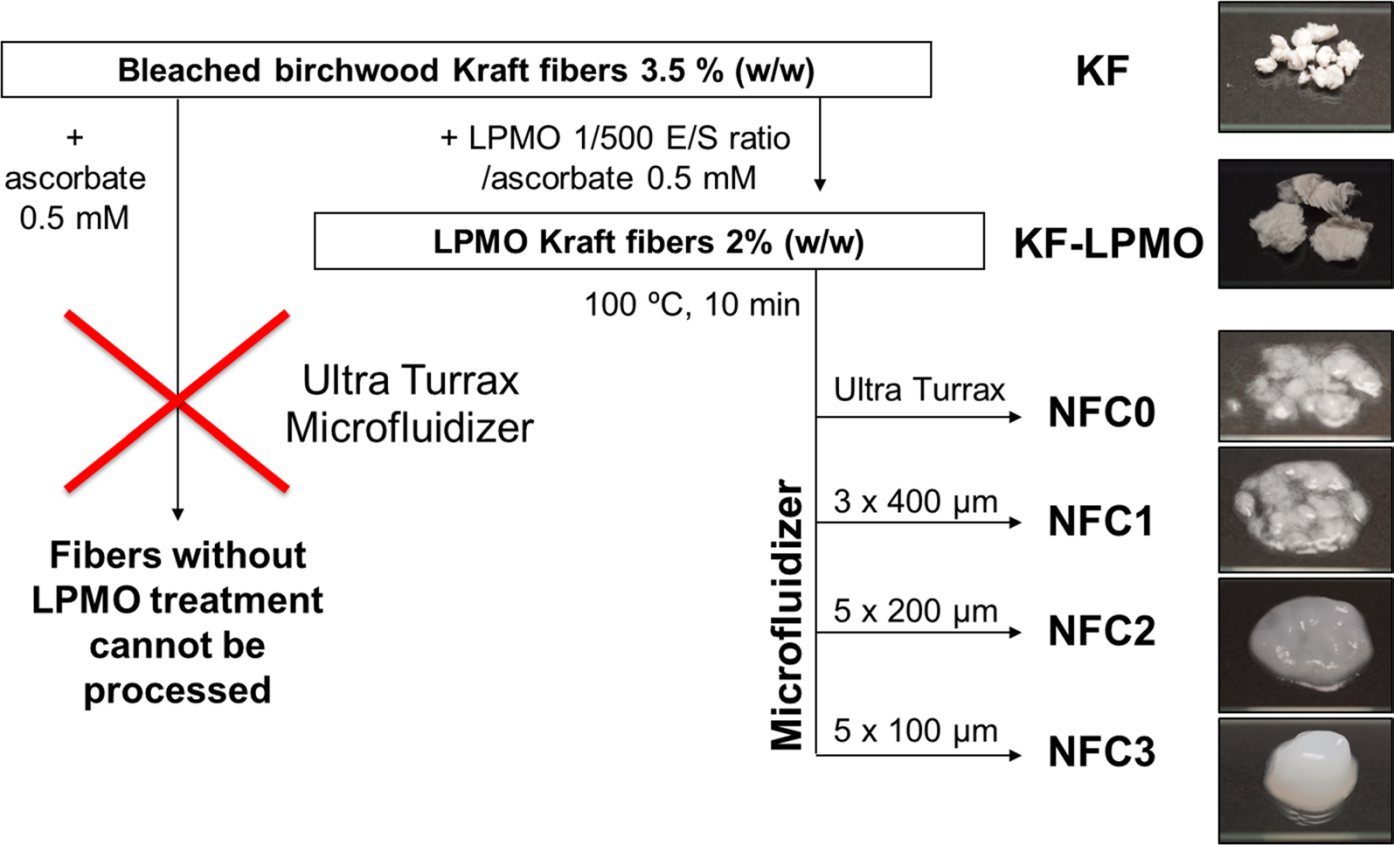
General scheme of the treatments performed on bleached birchwood Kraft fibers for the preparation of nanofibrillated cellulose (NFC) by *Pa*LPMO9E pretreatment combined with mechanical shearing and photographs of the samples

Nanofibrillated cellulose (NFC) was produced according to the work plan described in Fig. 1. Bleached birchwood Kraft fibers (28 g) at a consistency of 3.5% (w/w) were incubated with *Pa*LPMO9E and ascorbate as electron donor. Ascorbate was set at 0.5 mM, which is slightly lower in concentration than previously reported [30, 32]. However, taking into account the pulp consistency, the ascorbate/LPMO ratio is similar to our previous experiments. After 24 h of incubation, fibers were boiled for 10 min to inactivate the enzymes and then filtered and dispersed at 2% consistency (Additional file 1: Fig. S2). Firstly, supernatants of the *Pa*LPMO9E-treated fibers were analyzed by high performance anion exchange chromatography (HPAEC) for the detection of soluble non-oxidized and oxidized oligosaccharides released upon enzymatic action. Oxidized oligomers were detected, and the degree of polymerization (DP) ranged between DP2 and DP6 (Additional file 1: Fig S3), as previously observed for the action of the enzyme *Pa*LPMO9E on phosphoric acid swollen cellulose (PASC) [32]. Only C1-oxidized oligomers and not C4 oxidation products were detected confirming that *Pa*LPMO9E is active on cellulose substrate with a C1-type regioselectivity. After filtration and mild dispersion by an Ultra Turrax device, higher mechanical shearing was achieved by using a microfluidizer processor, which is commonly used for NFC production at industrial scale. The microfluidizer is equipped of three Z-chambers that progressively decrease in diameter (400, 200 and 100 μm) to increase shearing and fibrillation efficiency. The first significant result of this study was that LPMO-pretreated fibers were able to be processed in the microfluidizer without further mechanical pretreatment, which is the first requirement for the production of NFC from cellulose fibers. In contrary, control fibers that had undergone pretreatment in the same conditions but without enzyme loading could not be homogenized, as they blocked the system at the entrance to the cell. Figure 1 shows the photographs of bleached birchwood Kraft fibers throughout the treatments. LPMO-treated suspensions obtained after passing through the 200 µm chamber (NFC2) displayed gel consistency, which is an indirect proof of efficient dispersion at nanoscale. This effect was more pronounced in the case of NFC3 (passing through the 100 µm chamber).

### LPMO-treated fibers are efficiently nanofibrillated by microfluidization

**Fig. 2:**
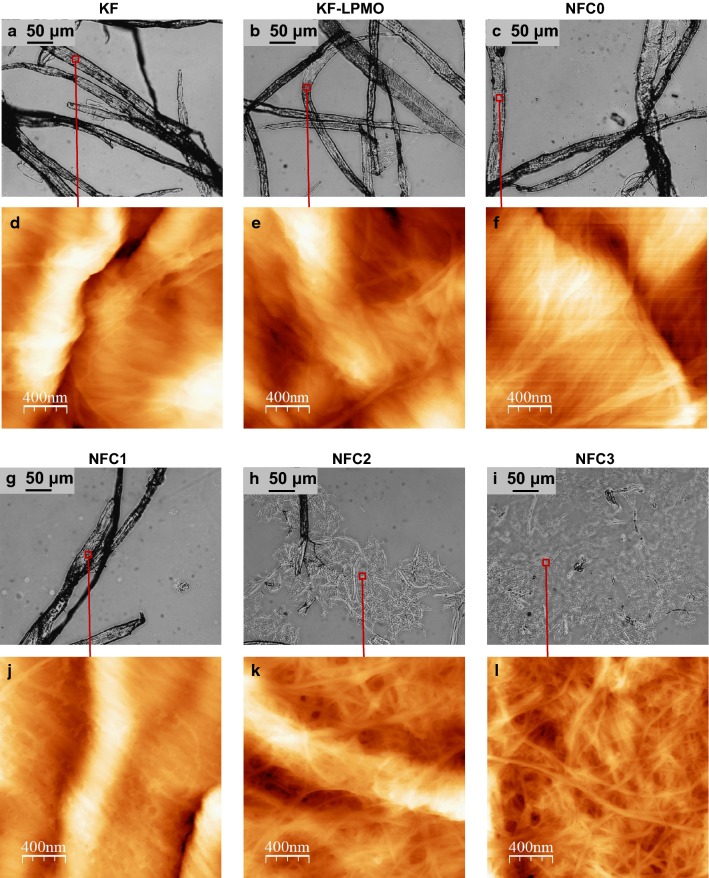
**a**–**c**, **g**–**i** Optical microscopy and **d**–**f**, **j**–**l** AFM images of starting bleached birchwood Kraft fibers (KF), *Pa*LPMO9E-treated bleached birchwood Kraft fibers (KF-LPMO), Ultra Turrax dispersed *Pa*LPMO9E-treated fibers (NFC0) and *Pa*LPMO9E-treated fibers submitted to mechanical shearing (NFC1–3). The red square in optical microscopy images indicates the area scanned by AFM

Optical microscopy images from starting bleached birchwood Kraft fibers mostly consisted of dispersed fibers of tens of micrometers in width and several hundreds of micrometers in length, which are the typical dimensions expected (Fig. 2). Upon *Pa*LPMO9E treatment, fibers still remained as bundles that seemed to be slightly fibrillated even if width did not change significantly. After microfluidization, fibrillation was clear for all samples. The first passes in the Z chamber of 400 μm (NFC1) produced the rupture of several bundles resulting in a slight decrease in width (13.4 ± 4.4 mm) and the release of small fragments that were scarcely detected by optical microscopy. The second passes through the Z chamber of 200 μm (NFC2) resulted in a clear decrease in both width and length of fibers, so that bundles were separated and cut in length. This effect was even more noticeable after the third treatment through the 100 μm chamber (NFC3), which produced a clear disruption of fibers. In this case, sample was completely fibrillated and a homogeneous gel-like matrix could be observed. Observations at the nanoscale by AFM supported these assumptions (Fig. 2). Starting birchwood Kraft fibers (KF) showed the particular pattern of wrinkles and microfibrils more or less aligned, characteristic of cellulose fibers [33]. After *Pa*LPMO9E treatment (KF-LPMO), AFM images showed a slight separation of the microfibrils, even if the fiber structure remained preserved. The effect of *Pa*LPMO9E was confirmed by the AFM images after dispersion by Ultra Turrax (NFC0), where more defined microfibrillated structures were observed. Even if optical microscopy images did not detect significant changes in the fiber structure, AFM suggested that *Pa*LPMO9E action resulted in the separation of microfibrils. When samples were submitted to the first shearing mechanical treatment (400 μm, NFC1), the fiber structure was still maintained; however, the passage through the 200 μm chamber (NFC2) provoked the disintegration of the fiber, with the subsequent release of nanofibrillated cellulose. This effect was more pronounced after the passage through the 100 μm chamber (NFC3), where the images clearly showed an entangled network of nanofibrillated cellulose.

**Fig. 3:**
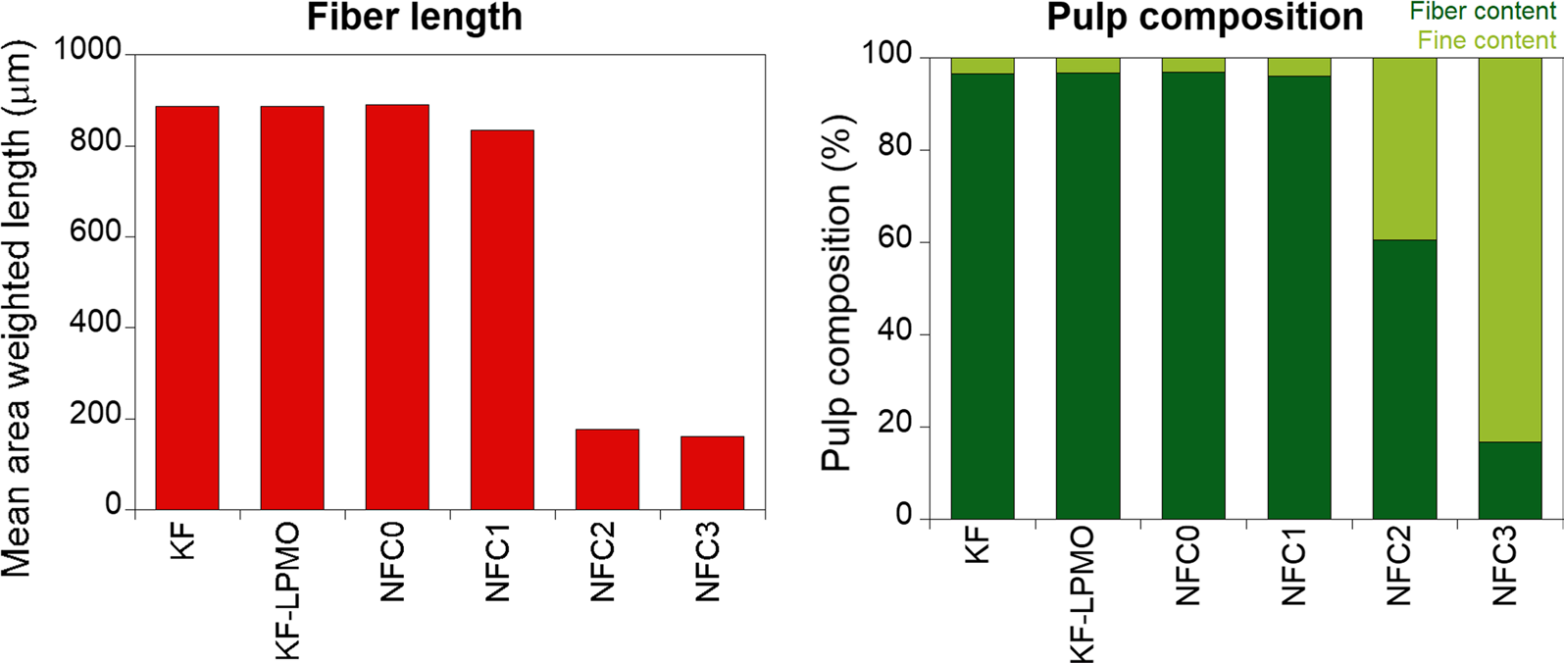
Geometrical characteristics (mean area-weighted length and percentage of fine content) of starting bleached birchwood Kraft fibers (KF), *Pa*LPMO9E-treated bleached birchwood Kraft fibers (KF-LPMO), Ultra Turrax dispersed *Pa*LPMO9E-treated fibers (NFC0) and *Pa*LPMO9E-treated fibers submitted to mechanical shearing (NFC1–3)

Optical and AFM images provided useful information but could be incomplete since they address only a portion of the sample. Thus, global assessment of the distribution pattern in the samples was determined by the MorFi analyzer. This analysis, with a detection threshold of 15 µm, does not characterize the smallest generated elements and nanoparticles but provides a size distribution of the residual fiber elements. The mean area-weighted length (Fig. 3) did not change significantly upon *Pa*LPMO9E treatment, or even after the passage through the 400 μm vessel (NFC1), while suspensions obtained after passing by the 200 µm vessels were highly microfibrillated (NFC2), as their gel consistency suggested. Then, higher fibrillation was obtained by performing a series of homogenization in the 100 µm chamber (NFC3). However, if we compare to similar treatments using endoglucanases or exoglucanases treatments at 0.1–1% loading followed by mechanical shearing, the lengths of fibers and the percentages of fine elements obtained in this work were still very coarse [34]. Usually, for the same working pulp pretreated by mechanical enzymatic pretreatment and homogenized with a 100 µm vessel, the suspensions have ~ 90% of fine elements (elements of size less than 80 µm) compared to 83% in the case of the pulp pretreated by LPMOs (NFC3).

### Monosaccharide composition is not affected by LPMO and mechanical treatments

The effect of both *Pa*LPMO9E and mechanical treatments was assessed by the determination of the monosaccharide composition of the samples throughout the process. Sample compositions are reported in Table 1.

**Table 1:** Monosaccharide composition on the percent dry weight basis of starting bleached birchwood Kraft fibers (KF), *Pa*LPMO9E-treated bleached birchwood Kraft fibers (KF-LPMO), Ultra Turrax dispersed *Pa*LPMO9E-treated fibers (NFC0) and *Pa*LPMO9E-treated fibers submitted to mechanical shearing (NFC1–3)

Sample	Rha	Ara	Xyl	Man	Glc	Inacc. cell
KF	0.5 ± 0.0	0.2 ± 0.1	23.7 ± 0.5	1.6 ± 1.0	74.1 ± 0.6	70.2 ± 1.0
KF-LPMO	0.4 ± 0.1	0.1 ± 0.0	23.8 ± 0.6	1.2 ± 0.5	74.4 ± 0.3	70.3 ± 0.3
NFC0	0.3 ± 0.1	0.2 ± 0.2	23.6 ± 0.1	1.1 ± 0.1	74.8 ± 0.3	70.1 ± 0.2
NFC1	0.4 ± 0.0	0.5 ± 0.1	23.9 ± 0.3	1.2 ± 0.0	74.0 ± 0.4	69.0 ± 0.6
NFC2	0.1 ± 0.1	0.3 ± 0.2	23.2 ± 0.3	0.8 ± 0.1	75.6 ± 0.5	69.6 ± 0.5
NFC3	0.4 ± 0.2	0.2 ± 0.2	23.1 ± 0.2	1.2 ± 0.3	75.2 ± 0.4	66.8 ± 0.4

Results are expressed as the mean percentage ± standard deviation

The major sugars recovered were glucose, xylose and small amounts of arabinose, galactose and rhamnose, as expected for hardwood delignified fibers containing cellulose and xylan as major polymers [35]. The major conclusion from the sugar analysis was that the monosaccharide profile did not change along the fractionation process neither after the *Pa*LPMO9E treatment nor during microfluidization. Other useful information that can be obtained from the monosaccharide determination assay is the amount of accessible and inaccessible cellulose fractions. Indeed, samples can be submitted either to a harsh hydrolysis that releases the total monosaccharides or to a milder acid hydrolysis that is usually recognized to release only the accessible sugar fractions (i.e., mostly from hemicellulose and amorphous cellulose) [36]. Even though this analysis remains questionable for the real structural features of polymers released, it gives valuable qualitative information in the case of samples submitted to stepwise modification such as in the present case. From the results, it appeared that the inaccessible fraction proportions (Table 1, last column) remained stable for the first three samples (KF, KF-LPMO and NFC0), suggesting that the *Pa*LPMO9E treatment did not change the major architecture of the fibers and likely the crystalline organization. During the mechanical treatment (NFC1–3), the values decreased slightly probably due to nanofibrillation and associated higher specific surface. Nevertheless, this decrease remained limited suggesting the retention of high crystallinity, which will be later confirmed by solid-state NMR.

### Molar mass distribution of cellulose chains is decreased by the mechanical treatment

**Fig. 4:**
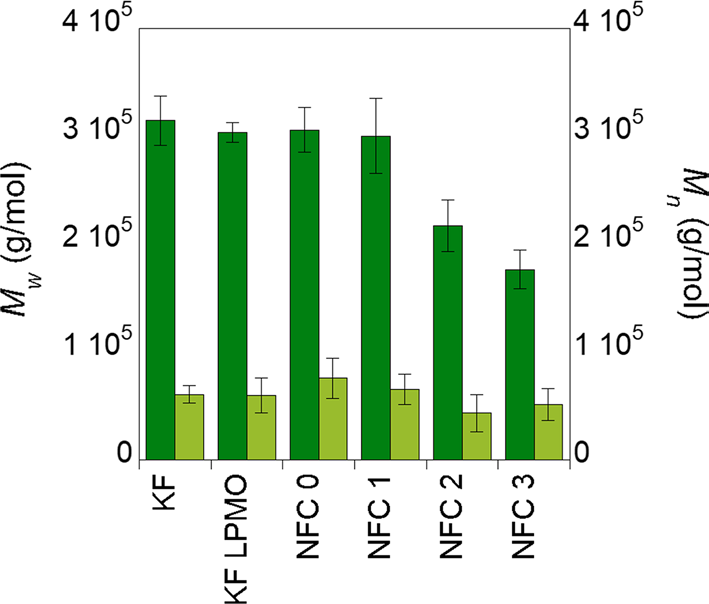
Weight average molar mass (*M*_w_) and number average molar mass (*M*_n_) of starting bleached birchwood Kraft fibers (KF), *Pa*LPMO9E-treated bleached birchwood Kraft fibers (KF-LPMO), Ultra Turrax dispersed *Pa*LPMO9E-treated fibers (NFC0) and *Pa*LPMO9E-treated fibers submitted to mechanical shearing (NFC1–3)

Dissolution of untreated and *Pa*LPMO9E-treated samples was successfully achieved by using the solvent system dimethyl acetamide (DMAc)/lithium chloride 9% (*w/w*) followed by elution in DMAc/LiCl 0.9%. The dissolution procedure used was inspired by the standardized protocol proposed by Potthast et al. [37], and the recovery of cellulose was in all the cases higher than 65% and even in some cases close to 85%, ensuring the reliability of the determination. Molar mass was determined by multi-angle laser light scattering (MALLS) as absolute molar mass determination detector and by a differential refractometer (RI) as concentration detector. The values of the weight average molar mass (*M*_w_) and number average molar mass (*M*_n_) and representative RI traces are reported in Fig. 4 and Additional file 1: Fig. S4, respectively. The values obtained for KF and KF-LPMO (i.e., samples before mechanical treatment) indicated that after the *Pa*LPMO9E treatment, only a very small decrease of the *M*_w_ was observed (315 10^5^ g mol^−1^ for KF vs 303 10^5^ g mol^−1^ for KF-LPMO) while the *M*_n_ values remained constant. This result seemed to indicate that *Pa*LPMO9E induced some cleavage of the cellulose chains but this observation needs to be strengthened by future studies since the difference observed was lower than the dispersion of the values. Concerning the mechanically treated samples, *M*_w_ values of NFC0 and NFC1 samples were identical to KF-LPMO (lower than KF) while the NFC2 and NFC3 showed a clear decrease. In all cases, *M*_n_ values remained stable within the error bar interval. The recovery yield decreased along the treatment. Hence, it ranged about 85% for KF, then decreased around 70% for KF-LPMO, NFC0 and NFC1 and finally reached 60–65% for the two last samples (NFC2 and NFC3). This fact could indicate the formation of nanometric elements that might be removed during the solvent exchange procedure or that display limited solubility as previously demonstrated [38].

### LPMOs trigger elementary fibrils separation but do not decrease the nanofibers crystallinity

**Fig. 5:**
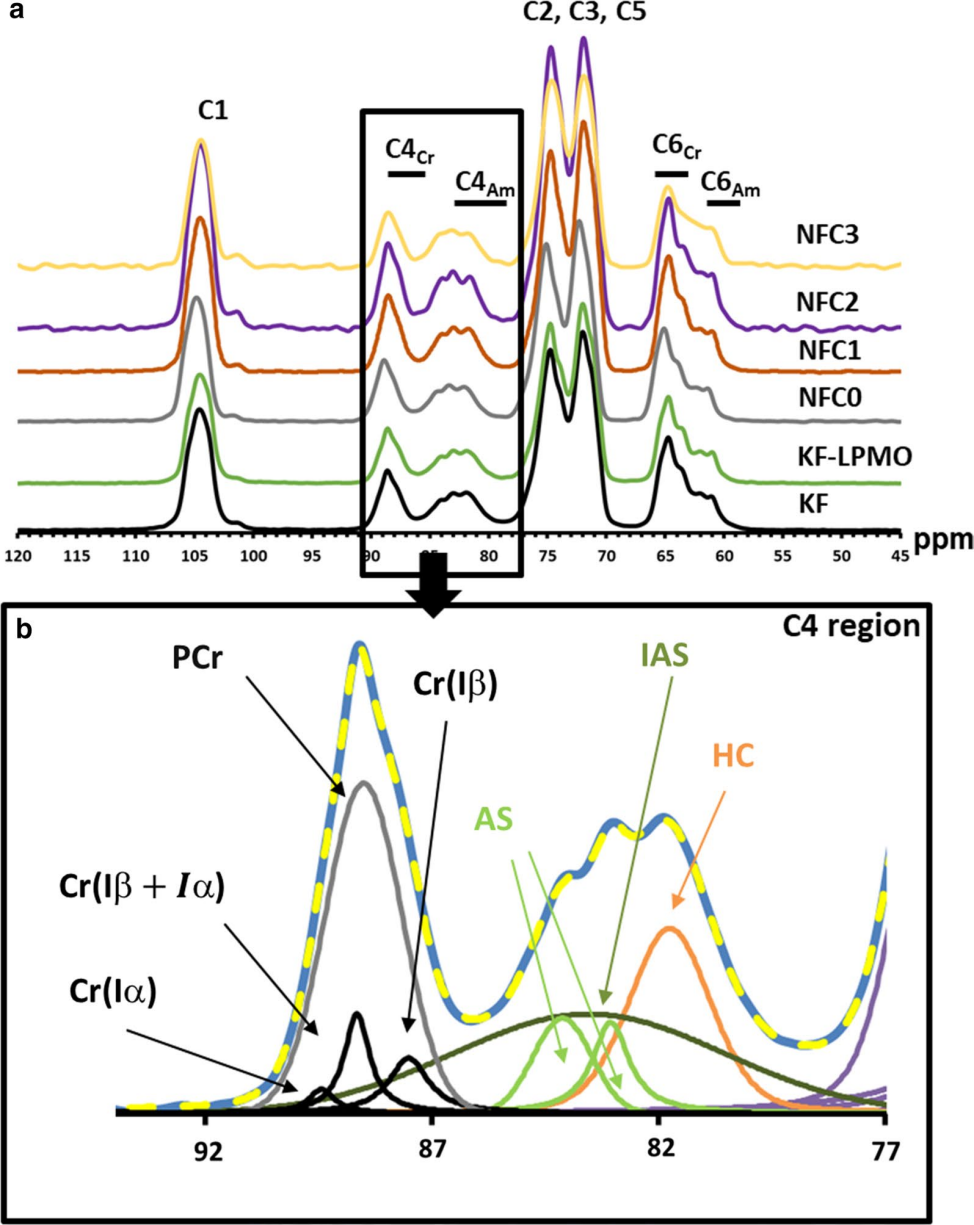
**a** Solid-state ^13^C CP/MAS NMR spectra of starting bleached birchwood Kraft fibers (KF, black line), *Pa*LPMO9E-treated bleached birchwood Kraft fibers (KF-LPMO, green line), Ultra Turrax dispersed *Pa*LPMO9E-treated fibers (NFC0, gray line) and *Pa*LPMO9E-treated fibers submitted to mechanical shearing by the 400 μm chamber (NFC1, red line), by the 200 μm chamber (NFC2, purple line), and by the 100 μm chamber (NFC3, yellow line). The frame part of the NMR spectra corresponds to the C4 region. **b** Typical deconvolution of the C4 region from ^13^C CP/MAS NMR spectrum of the KF sample. Cr(Iα), Cr (Iβ) and Cr (Iα + β) correspond to crystalline cellulose forms; PCr corresponds to para-crystalline contribution; AS/IAS corresponds to accessible/inaccessible surfaces in the amorphous part of cellulose; and HC to hemicellulose contribution

To investigate deep changes induced in the fiber structure, solid-state ^13^C CP/MAS NMR spectroscopy was used to analyze cellulose fibers before and after the *Pa*LPMO9E action and mechanical treatments. The different regions of the ^13^C CP/MAS NMR spectra show the typical distinct signals of C1 (*δ* 98–108 ppm), C4 (*δ* 78–92 ppm), C2, C3, C5 carbons (*δ* 68–78 ppm) and C6 (*δ* 58–68 ppm) from cellulose (Fig. 5a). The most informative region in the NMR spectra of cellulose is the C4 region between 78 and 92 ppm, which contains sharp signals ranging from 86 to 92 ppm corresponding to C4 carbons situated in crystalline cellulose domains (C4_Cr_ in Fig. 5a). The C4 carbons of more disordered regions (or amorphous domains) are distributed in a broad band ranging from 78 to 86 ppm (C4_am_, Fig. 5a). The ^13^C CP/MAS spectra of all samples were deconvoluted by fitting C4-region according to the NMR fitting procedure previously reported [30, 39, 40]. Typically, fitted lines of the C4 (δ 78–92 ppm) are shown in Fig. 5b with four peaks, Cr(Iα), Cr (Iβ) and Cr (Iα + β) corresponding to crystalline cellulose forms, together with para-crystalline (PCr) cellulose. For the non-crystalline cellulose domain, three peaks were detected: a pair of signals at 83 and 84 ppm assigned to two non-equivalent sites at the accessible crystallite surfaces (AS) and a very broad signal at 83.4 ppm assigned to inaccessible cellulose surface (IAS). The broad peak located at ~ 81.4 ppm in the C4 region was assigned to hemicellulose and more specifically to xylan in accordance with the monosaccharide composition. From the fitting data, the accessible (AS)/total fibril (AS + IAS) surface ratio was determined for all samples as well as the cellulose crystallinity index (CrI) defined as the peak area ratio of four lines for the crystalline part (i.e., Cr(Iα), Cr (Iβ) and Cr (Iα + β) and PCr) and seven lines for all the cellulose C-4 region [40, 41]. All results are summarized in Table 2.

**Table 2:** Values of crystallinity index (CrI), hemicellulose percentage (%HC), lateral fibril dimensions (LFD) and lateral fibril aggregate dimensions (LFAD) and accessible/total fibril surface ratio (AS/(AS + IAS)), calculated from the C4-region deconvolution of the solid-state ^13^C CP/MAS NMR spectra from the starting bleached birchwood Kraft fibers (KF), *Pa*LPMO9E-treated bleached birchwood Kraft fibers (KF-LPMO), Ultra Turrax dispersed *Pa*LPMO9E-treated fibers (NFC0) and *Pa*LPMO9E-treated fibers submitted to mechanical shearing (NFC1–3)

Sample	CrI (%)	HC (%)	LFD^a^ (nm)	LFAD^a^ (nm)	AS/ (AS + IAS)
KF	50.28 ± 2.99	22.42 ± 2.39	2.27 ± 0.13	19.05 ± 4.43	24.18 ± 4.38
KF-LPMO	49.94 ± 1.54	18.97 ± 2.23	2.20 ± 0.07	18.00 ± 2.18	24.86 ± 2.29
NFC0	51.13 ± 0.93	19.12 ± 0.52	2.23 ± 0.93	15.14 ± 0.42	29.67 ± 0.42
NFC1	53.71 ± 0.02	19.82 ± 1.35	2.12 ± 0.06	14.69 ± 2.39	32.77 ± 2.39
NFC2	47.88 ± 0.02	18.85 ± 2.34	2.39 ± 0.12	11.29 ± 0.40	36.73 ± 0.40
NFC3	47.65 ± 0.80	20.19 ± 1.17	2.39 ± 0.03	9.33 ± 0.43	43.86 ± 0.43

^a^Assuming a factor of 0.57 nm

The degree of cellulose crystallinity was similar to previous values obtained for pulps of birch containing hemicelluloses [42]. Concerning the *Pa*LPMO9E and mechanical treatments, CrI index was found to be rather stable for all samples. No change was observed after the *Pa*LPMO9E treatment, and only a slight reduction was observed after the passage through the 200 μm chamber (NFC2). The hemicellulose content was also determined, and results were consistent with the monosaccharide composition; it was found to be stable around 20% suggesting that neither the *Pa*LPMO9E action nor the mechanical disintegration induced a hemicellulose removal. Lateral fibril (LFD) and fibril aggregate (LFAD) dimensions were also determined [43]. Concerning LFD, calculated values were also found stable at about 2.2 nm, slightly lower than values reported for other cellulosic fibers [30, 43, 44]. In contrary, LFAD decreased during the treatment from 19 nm, found for starting birchwood fibers, down to 9 nm for the *Pa*LPMO9E-treated and mechanically disintegrated (NFC3) samples. Nevertheless, the major decrease was observed during the mechanical treatment and not after the *Pa*LPMO9E action, and it was more remarkable as the chamber diameter was reduced. Therefore, the accessible surface increased during all treatments, which confirmed the separation of fibrils at nanoscale. This finding was in good agreement with AFM images that showed mechanical delamination and the formation of individual nanofibers upon the effect of *Pa*LPMO9E and mechanical shearing.

## Discussion

### LPMO action allows efficient nanofibrillation of cellulosic fibers

In this work, we used a monocomponent LPMO enzyme system as a pretreatment of cellulose fibers for the NFC production. We submitted bleached Kraft pulp fibers to the action of the fungal *Pa*LPMO9E and further mechanical treatment by microfluidizer. Currently, for any production of NFCs from bleached Kraft fibers, it is necessary to carry out a pretreatment prior to homogenization in order to reduce the fibers size and to allow them to pass through the homogenizer without clogging the chambers. Presently, at industrial scale, the most commonly used pretreatments are chemical or enzymatic. Chemical pretreatments, such as TEMPO-mediated oxidation or carboxymethylation, introduce negative charges on the fiber surface inducing electrostatic repulsion that improves the disintegration of cellulose fibers [18, 19, 45]. Enzymatic pretreatments are usually achieved by the action of endoglucanases either alone or in synergy with other enzymes in order to facilitate the refining process treatment [18–25].

Lytic polysaccharide monooxygenase enzymes appear as a novel strategy for facilitating fibrillation of cellulose fibers. In previous studies, we had demonstrated that the enzyme *Pa*LPMO9H induces modifications on the cellulose network that trigger the disintegration of fibers and facilitate the formation of nanofibers [30, 46]. Even using a mild mechanical treatment consisting of dispersion and sonication, cellulose fibers were disrupted down to nanoscale. Subsequent studies performed by Valenzuela et al. [28] investigated the synergy between bacterial LPMOs and endoglucanases followed by high-pressure homogenization for the production of NFC from flax pulp. The authors found significantly higher yields when both enzymes were combined (24.3%) compared to pure LPMO (12.7%) or monocomponent endoglucanase (17.0%) alone. Yields are far from that reported for chemical pretreatments of flax fibers, such as TEMPO at a concentration of 5 mg per gram of cellulose (yield about 70%) [47, 48], which could be ascribed to the content of hemicelluloses that hinder the fibrillation process. Hu et al. [29] used a combination of endoglucanase, LPMO and xylanases to enhance nanofibrillation of bleached Kraft pulp. The authors demonstrated that the addition of xylanase favors the cleavage of cellulose chains by LPMO and endoglucanases [49].

In this work, the first and most important result is that the *Pa*LPMO9E-treated fibers can be efficiently processed without any clogging of the devices while the reference fibers that were not *Pa*LPMO9E-treated blocked the microfluidizer. Even if no change is observed in size and morphology of birchwood fibers after *Pa*LPMO9E treatment, mechanical shearing clearly revealed the efficient action of *Pa*LPMO9E. The passage through the 400 μm chamber produced a slight decrease in fiber length; however, it was the passage through 200 μm which induced fibrillation of the fibers and a global decrease of length and diameter. Similarly to common enzymatic treatments (endoglucanase and/or exoglucanase), pure *Pa*LPMO9E disrupted birchwood fibers to the nanometric size, as shown using AFM. Siqueira et al. [34] observed that exoglucanases preserved the network structure of cellulose, whereas the action of endoglucanases resulted in a mixture of nanofibers and nanocrystals. In our study, the action of *Pa*LPMO9E yielded a pulp with a rather uniform nanofibril composition that potentially reduces the need for mechanical refining [50].

### LPMO action mechanism assumption

Beside the demonstration of the efficiency of *Pa*LPMO9E to produce NFC, the aim of our work was also to provide new insights in the LPMO mechanism of action on the cellulosic fibers. The fibers structure was thus investigated by various techniques leading to the following three main features: (i) *Pa*LPMO9E cleaved the cellulosic chains as demonstrated by the HPAEC results. Nevertheless, the cleavage was somehow limited since the amount of oxidized oligomers was low. HPSEC analysis led to similar conclusions since a small decrease in molar mass was observed after the *Pa*LPMO9E treatment for KF-LPMO, NFC0 and NFC1 samples, even if these observations need to be further investigated. Nevertheless, this small variation could be associated with a decrease in the recovery yield. One possible explanation is the formation of nanometric fractions that might be lost during solvent exchange, suggesting that the *Pa*LPMO9E action occurred in the outer surface of the fibers protecting the inner cellulosic chains. (ii) *Pa*LPMO9E did not directly induce nanofibrils disruption since all microscopy techniques and ^13^C CP/MAS NMR indicated that nanofibrillation occurred when the diameter of the Z chamber was reduced to 200 μm and not before (e.g., not after the *Pa*LPMO9E treatment). HPSEC data confirmed this trend since a drastic decrease of *M*_w_ was observed for the NFC2 sample compared to NFC1, indicating that the weakening of the fibers boosts mechanical action to reduce the chain length. (iii) *Pa*LPMO9E did not change the fibers crystallinity as indicated directly by the ^13^C CP/MAS NMR results and indirectly by the monosaccharide composition.

The combination of these results leads to the conclusion that *Pa*LPMO9E likely acts at the surface of the elementary fibril through an oxidative cleavage that releases only few short soluble oligomers (lower than 0.1% of the fibers mass), while the major part of the chains remains stacked together. In fact, in the model of cellulose arrangement [43], cellulose fibrils are mainly associated as a part of aggregates, which means that a high proportion of the fibril surface area and crystallinity regions are inside aggregates, preventing direct enzymatic attack. Thus, the inner part of the elementary fibers seems to be preserved after *Pa*LPMO9E action keeping crystallinity and molar mass nearly intact. Nevertheless, mechanical treatment provides enough energy to disrupt the *Pa*LPMO9E-treated cellulose network inducing a clear weakening of the elementary fiber cohesion and increasing the accessible surface.

## Conclusions

This work describes for the first time the action of a single LPMO followed by mechanical shearing as a strategy for the fabrication of nanofibrillated cellulose (NFC). The treatment of birchwood fibers by *Pa*LPMO9E alone did not modify fiber cohesion and morphology, and fiber dimensions seemed to remain unchanged before mechanical treatment. Nevertheless, the use of mechanical shearing by a microfluidizer processor induced fibrillation and nanometer-sized fibrils were obtained. The use of LPMO enzymes not only allowed the passage of cellulose fibers through the microfluidizer chamber, but also resulted in a homogeneous distribution of nano-size fibrils that maintained fiber crystallinity. This enzymatic process is upscalable as the production of recombinant LPMO was set up in bioreactor.

## Materials and methods

### *Pa*LPMO9E production

The codon-optimized DNA sequence (Genebank ID CAP67740) encoding was previously synthesized as described in Bennati-Granier et al. [32]. In this study, an alternative construct where the yeast α-MF from the expression vector pPICZ-α was swapped with the native signal peptide was designed. The *Pme*I-linearized plasmid was used for transformation into electrocompetent *P. pastoris* X33 cells as described previously [51]. Zeocin-resistant *P. pastoris* transformants were then screened for optimal protein production. The best-producing transformants were grown in 1 L of BMGY containing 1 mL L^−1^ of PTM_4_ salts (2 g L^−1^ CuSO_4_·5H_2_O; 3 g L^−1^ MnSO_4_·H_2_O; 0.2 g L^−1^ Na_2_MoO_4_·2H_2_O; 0.02 g L^−1^ H_3_BO_3_; 0.5 g L^−1^ CaSO_4_·2H_2_O; 0.5 g L^−1^ CoCl_2_; 12.5 g L^−1^ ZnSO_4_·7H_2_O; 22 g L^−1^ FeSO_4_·7H_2_O; biotin 0.2 g L^−1^; concentrated H_2_SO_4_ 1 mL) in shaken flasks at 30 °C in an orbital shaker (200 rpm) for 16 h to an OD_600_ of 2–6. Expression was induced by transferring the cells into 200 mL of BMMY containing 1 ml L^−1^ of PTM_4_ salts at 20 °C in an orbital shaker (200 rpm) for another 3 days. Each day the medium was supplemented with 3% (v/v) methanol.

Bioreactor production of *Pa*LPMO9E was carried out in 1.3-L New Brunswick BioFlo 115 fermentors (Eppendorf, Hamburg, Germany) following the *P. pastoris* fermentation process guidelines (Invitrogen) as described in Couturier et al. [39]. Recombinant enzyme was secreted up to ~ 2 g L^−1^ (Additional file 1: Figs. S1, S2).

### Purification of *Pa*LPMO9E

The culture supernatants were recovered by pelleting the cells by centrifugation at 2700*g* for 5 min, 4 °C and filtered on 0.45 µm filters (Millipore, Molsheim, France) to remove any remaining cells. After adjusting the pH to 7.8, the supernatants were filtered once more on 0.2 µm filters and loaded onto 5 mL Histrap columns (GE healthcare, Buc, France) connected to an Akta Xpress system (GE healthcare). Prior to loading, the columns were equilibrated in Tris HCl 50 mM pH 7.8 and NaCl 150 mM (buffer A). The loaded columns were then washed with five column volumes (CV) of 10 mM imidazole in buffer A, before the elution step with 5 CV of 150 mM imidazole in buffer A. After elution, the fractions containing the purified proteins were pooled and buffer was exchanged to Tris HCl pH 7.8, NaCl 50 mM using PD-10 columns (GE Healthcare). An aliquot of each fraction was loaded onto an SDS-PAGE stain-free gel (Bio-rad, Marnes-la-Coquette, France) to check protein purity. Protein concentration was determined with a Nanodrop ND-2000 spectrophotometer (Thermo Fisher Scientific, IL, USA) using the theoretical mass and the molar extinction coefficient calculated from the protein sequence.

### Cellulosic fibers

A commercial bleached birchwood Kraft pulp from Stora Enso was used.

### LPMO treatment and NFC production

All procedure steps of bleached birchwood Kraft fibers (KF) through enzymatic treatment and homogenization procedures are summarized in Fig. 1. Enzymatic treatment of cellulosic fibers (28 g in total) with *Pa*LPMO9E was carried out in a Tornado multiple chamber reactor (Additional file 1: Fig. S2). Enzymatic treatment was run in parallel in four reaction chambers in order to assess reproducibility. Enzymatic reaction was performed in sodium acetate buffer (50 mM, pH 4.8) in the presence of ascorbic acid at 0.5 mM using an enzyme/substrate ratio of 1:500 with a 3.5% (*w/v*) consistency during 24 h under constant stirring and at 50 °C. The enzymatic reaction was stopped by boiling for 10 min, and the wet cake was obtained by filtration and washing (Additional file 1: Fig. S2). Fibers were redispersed at 2% concentration (*w/w*) in water (KF-LPMO). The suspensions obtained were homogenized using an Ultra Turrax T25 homogenizer for 30 s at 7500 rpm (short time to improve fiber dispersion without cutting) (NFC0). The suspensions obtained were then homogenized through a M-110 EH-30 microfluidizer processor (Microfluidics, USA) at a concentration of 2% (*w/w*, dry matter content). The slurry was passed through a piston pump that applies a high pressure. This microfluidizer has three Z-shaped interaction chambers with internal diameters of 400, 200 and 100 µm, and it allows working at a constant flow rate of about 350 mL min^−1^. Pulp suspension passed first three times through the 400 µm chamber with operating pressure of 100 bar (NFC1), then five times through a chamber of 200 µm, where the operating pressure was 1500 bar (NFC2), and finally five times through the 100 µm chamber operating at 2000 bar (NFC3).

### Analysis of the soluble sugars by HPAEC-PAD

The oxidized and non-oxidized oligosaccharides generated after action of *Pa*LPMO9E on bleached birchwood Kraft fibers were analyzed by high-performance anion exchange chromatography coupled with pulsed amperometric detection (HPAEC-PAD) (Thermo Fisher Scientific, Waltham, USA) as described by Westereng et al. [52] using non-oxidized cello-oligosaccharides as standards (Megazyme, Wicklow, Ireland). Oligosaccharides standards oxidized at the C1 position were produced from non-oxidized cello-oligosaccharides using a cellobiose dehydrogenase as described in Bennati-Granier et al. (31).

### Morphological analysis of the fibers

The morphological characteristics of the fibers contained in the suspensions (starting bleached birchwood Kraft fibers or LPMO-pretreated fibers) were determined by passing through the MorFi analyzer (Techpap, France), based on optics and flow cell measurement.

### Optical and atomic force microscopy

Cellulose fibers were deposited onto freshly cut mica substrates from fiber solutions at 0.1 g L^−1^ and dry overnight. Fibers were observed by an Olympus IX51 microscope with a 20× objective. Atomic force microscopy imaging was performed with the cantilever located on certain cellulose fibers with the aid of an optical microscope. Topographical images were registered by a Catalyst AFM (Bruker). The images were obtained in tapping mode under ambient air conditions (temperature and relative humidity) using a monolithic silicon tip (Scanasyst-Air, Bruker) with a spring constant of 0.4 N m^−1^ and a nominal frequency of 70 kHz. Image processing was performed with the WSxM 4.0 software [53].

### Monosaccharide composition determination

Individual neutral sugar composition of cellulosic fibers was identified and quantified after sulfuric acid degradation [36] as their alditol acetates derivatives by gas–liquid chromatography (GC) [54]. For total monosaccharides determination, 5 mg of dried cellulosic sample was firstly prehydrolyzed by 13 M sulfuric acid for 1 h at 30 °C and then hydrolyzed in 1 M sulfuric acid for 2 h at 100 °C. For accessible monosaccharide determination, soluble fractions were hydrolyzed by 2 M TFA at 120 °C for 2 h. GC analysis was performed with a TG-225 GC column (30 × 0.32 mm ID) using a TRACE™ Ultra Gas Chromatograph (Thermo Scientific^TM^; temperature 205 °C. carrier gas H_2_). Standard sugars solution and inositol as internal standard were used for calibration. All sample analyses were done in triplicate.

### High performance size exclusion chromatography (HPSEC)

About 80–100 mg of cellulose samples was weighted and dispersed in water (0.1% *wt*) during one night under vigorous stirring. Fibers were then filtered through 0.45 μm PTFE membranes. The fiber cake was then redispersed three times in anhydrous methanol (50 mL each time) followed by three additional redispersions in anhydrous dimethylacetamide (50 mL). Then, the DMAc fibers swollen cake was added to 5 or 10 mL of DMAc/LiCl (9% w/w) under mechanical stirring during 24 h before tenfold dilution with anhydrous DMAc. The solution was then filtered and injected on a size exclusion chromatography system (OMNISEC Resolve, Malvern) with *N*,*N*-dimethylacetamide/lithium chloride (0.9% *w/v*) as the eluent. The SEC columns used were Viscotec Tguard, LT4000L, LT5000L and LT7000L. The system was equipped with a multi-angle laser light scattering Malvern SEC-MALS 20 and OMNISEC Reveal devices (Malvern). Calculations were performed with a *dn/dc* value of 0.136 mL g^−1^ and performed using OMNISEC software.

### Cross-polarization/magic angle spinning (CP/MAS) NMR

For NMR analysis, samples (100 mg) were rehydrated in 50 µL H_2_O and water excess was absorbed using an adsorbent. About 80–100 mg of each sample was packed into 4 mm NMR rotor. All cross-polarization magic angle (CP/MAS) NMR experiments were acquired on a Bruker Avance III 400 spectrometer operating at a ^13^C frequency of 100.62 MHz equipped with a double-resonance H/X CP/MAS 4 mm probe. Measurements were conducted at room temperature with a MAS spinning rate of 9 kHz. The CP pulse sequence parameters were 3.5 μs proton 90° pulse, 1.75 ms CP contact time at 67.5 kHz and 9 s recycle time. The number of acquisitions for the CP/MAS ^13^C spectra was typically 5.120 scans. ^13^C NMR spectra were referenced to the carbonyl peak of glycine at 176.03 ppm. All spectra were processed with Gaussian multiplication parameters of LB = − 5 Hz and GB = 0.1.

From all NMR spectra, C4 regions were deconvoluted using Lorentzian lines for the crystalline part (Cr (Iα) and Cr (Iβ)) and one Gaussian line for the less ordered cellulose (para-crystalline cellulose, PCr, accessible surfaces, AS, and inaccessible surface, IAS) (see Additional file 1: Table S1 and Fig. 5) [30, 41]. Signal at 81.74 ppm was assigned to hemicellulose (HC), more specifically xylan, and was deconvoluted into one Gaussian line. The cellulose crystallinity, measured as the crystallinity index (CrI), was determined from the areas of the crystalline (*A*_cryst_, 86–92 ppm) and amorphous (*A*_amorp_, 78–86 ppm) C4 signals from spectral deconvolution as ${\text{CrI}} = A_{\text{crys}} /\left( {A_{\text{cryst}} + A_{\text{amorp}} } \right) \times 100\%$.

## Additional file

**Additional file 1: Fig S1.** Monitoring of the recombinant production of *Pa*LPMO9E in a 1.3 L bioreactor. **Fig S2 a** Image of the enzymatic treatment of birchwood Kraft fibers in the Tornado multiple chamber reactor. The enzymatic treatment was performed in parallel in four chambers using 7 g of cellulose fibers in each of them. **b** Aspect of the *Pa*LPMO9E-treated bleached birchwood Kraft fibers after filtration. **Fig S3 a** HPAEC chromatograms showing the elution profile of oxidized and non-oxidized oligosaccharides and **b** quantification by HPAEC analysis of the soluble sugars released by the action of *Pa*LPMO9E on starting bleached birchwood Kraft fibers (KF), *Pa*LPMO9E-treated bleached birchwood Kraft fibers (KF-LPMO), Ultra Turrax dispersed *Pa*LPMO9E-treated fibers (NFC0) and *Pa*LPMO9E-treated fibers submitted to mechanical shearing (NFC1–3). **Fig S4** Normalized HPSEC-RI chromatograms of solubilized fibers from starting bleached birchwood Kraft fibers (KF), *Pa*LPMO9E-treated bleached birchwood Kraft fibers (KF-LPMO), Ultra Turrax dispersed *Pa*LPMO9E-treated fibers (NFC0) and *Pa*LPMO9E-treated fibers submitted to mechanical shearing (NFC1–3). **Table S1.** Characteristics (chemical shift position of the peaks, δ, and full width at half height, FWHH) of signals generated from the deconvolution at the C-4 region of the solid-state ^13^C CP/MAS NMR spectra of starting bleached birchwood Kraft fibers (KF), *Pa*LPMO9E-treated bleached birchwood Kraft fibers (KF-LPMO), Ultra Turrax dispersed *Pa*LPMO9E-treated fibers (NFC0) and *Pa*LPMO9E-treated fibers submitted to mechanical shearing (NFC1–3).

## Data Availability

All data generated or analyzed during this study are included in this published article and its additional information files.

## Abbreviations

AFM: atomic force microscopy
CP/MAS: cross-polarization/magic angle spinning
HPAEC-PAD: high-performance anion exchange chromatography coupled with pulsed amperometric detection
HPSEC: high-performance size exclusion chromatography
KF: bleached birchwood Kraft fibers
KF-LPMO: bleached birchwood Kraft fibers treated by *Pa*LPMO9E
LPMO: lytic polysaccharide monooxygenase
MALLS: multi-angle laser light scattering
*M*_n_: number average molar mass
*M*_w_: weight average molar mass
NFC: nanofibrillated cellulose
NFC0: bleached birchwood Kraft fibers treated by *Pa*LPMO9E and submitted to Ultra Turrax dispersion
NFC1–3: bleached birchwood Kraft fibers treated by *Pa*LPMO9E and submitted to mechanical shearing
PASC: phosphoric acid swollen cellulose
RI: differential refractometer
TEMPO: 2,2,6,6-tetramethylpiperidine-1-oxyl
