# Supplementary material for: Lytic polysaccharide monooxygenases (LPMOs) facilitate cellulose nanofibrils production
Source: Biotechnol Biofuels. 2019 Jun 24;12:156. doi: 10.1186/s13068-019-1501-0 (PMC6589874; doi:10.1186/s13068-019-1501-0)
Supplement: Supplementary file 1 — Additional file 1: Fig S1. Monitoring of the recombinant production of PaLPMO9E in a 1.3 L bioreactor. Fig S2 a Image of the enzymatic treatment of birchwood Kraft fibers in the Tornado multiple chamber reactor. The enzymatic treatment was performed in parallel in four chambers using 7 g of cellulose fibers in each of them. b Aspect of the PaLPMO9E-treated bleached birchwood Kraft fibers after filtration. Fig S3 a HPAEC chromatograms showing the elution profile of oxidized and non-oxidized oligosaccharides and b quantification by HPAEC analysis of the soluble sugars released by the action of PaLPMO9E on starting bleached birchwood Kraft fibers (KF), PaLPMO9E-treated bleached birchwood Kraft fibers (KF-LPMO), Ultra Turrax dispersed PaLPMO9E-treated fibers (NFC0) and PaLPMO9E-treated fibers submitted to mechanical shearing (NFC1–3). Fig S4 Normalized HPSEC-RI chromatograms of solubilized fibers from starting bleached birchwood Kraft fibers (KF), PaLPMO9E-treated bleached birchwood Kraft fibers (KF-LPMO), Ultra Turrax dispersed PaLPMO9E-treated fibers (NFC0) and PaLPMO9E-treated fibers submitted to mechanical shearing (NFC1–3). Table S1. Characteristics (chemical shift position of the peaks, δ, and full width at half height, FWHH) of signals generated from the deconvolution at the C-4 region of the solid-state 13C CP/MAS NMR spectra of starting bleached birchwood Kraft fibers (KF), PaLPMO9E-treated bleached birchwood Kraft fibers (KF-LPMO), Ultra Turrax dispersed PaLPMO9E-treated fibers (NFC0) and PaLPMO9E-treated fibers submitted to mechanical shearing (NFC1–3). [file 13068_2019_1501_MOESM1_ESM.docx]

**Additional information**

# **Lytic Polysaccharide Monooxygenases (LPMOs) facilitate Cellulose Nanofibrils production**

Céline Moreau^1^, Sandra Tapin-Lingua^2^, Sacha Grisel^3^, Isabelle Gimbert^3^, Sophie Le Gall^1^, Valérie Meyer^4^, Michel Petit-Conil^4^, Jean-Guy Berrin^3^, Bernard Cathala^1^, Ana Villares^1^

^1^ BIA, INRA, 44300, Nantes, France.

^2^ FCBA, InTechFibres Division, Domaine Universitaire, CS 90252, 39044 Grenoble Cedex 9, France

^3^ Biodiversité et Biotechnologie Fongiques, INRA, Aix Marseille University, UMR1163,

13009, Marseille, France.

^4^ CTP, Domaine Universitaire, CS 90252, 39044 Grenoble Cedex 9, France

***Production of* Pa*LPMO9E in bioreactor***

**Fig S1** Monitoring of the recombinant production of *Pa*LPMO9E in a 1.3 L bioreactor. OD, optical density; DO, dissolved oxygen

***LPMO pre-treatment allow the production of NFC***


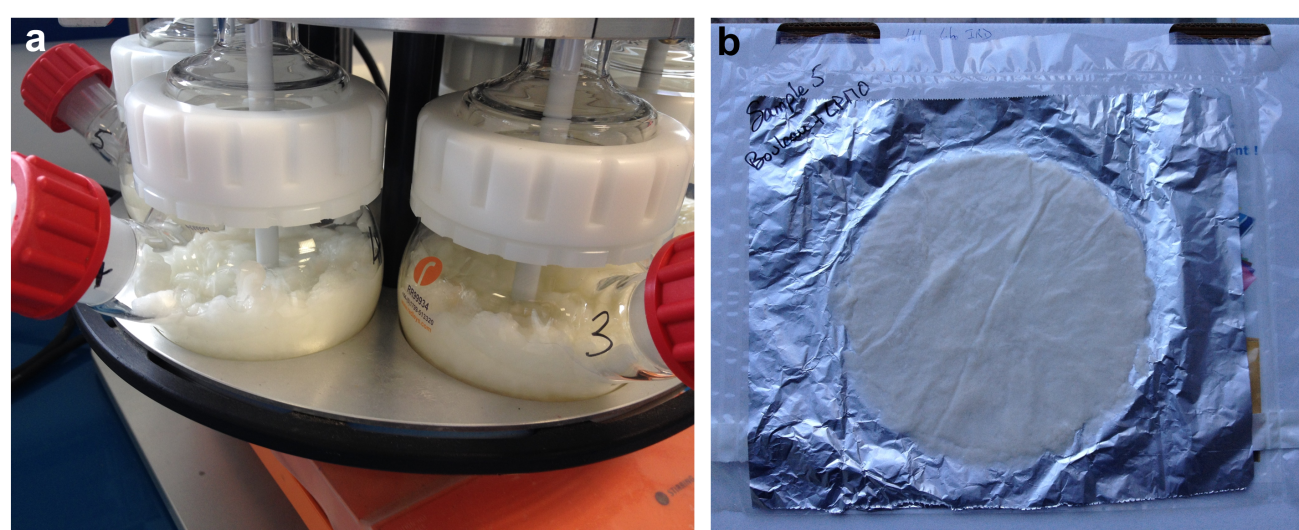


**Fig S2** **a** Image of the enzymatic treatment of birchwood Kraft fibers in the Tornado multiple chamber reactor. The enzymatic treatment was performed in parallel in four chambers using 7 g of cellulose fibers in each of them. **b** Aspect of the *Pa*LPMO9E-treated bleached birchwood Kraft fibers after filtration

***LPMO pre-treatment allow the production of NFC***


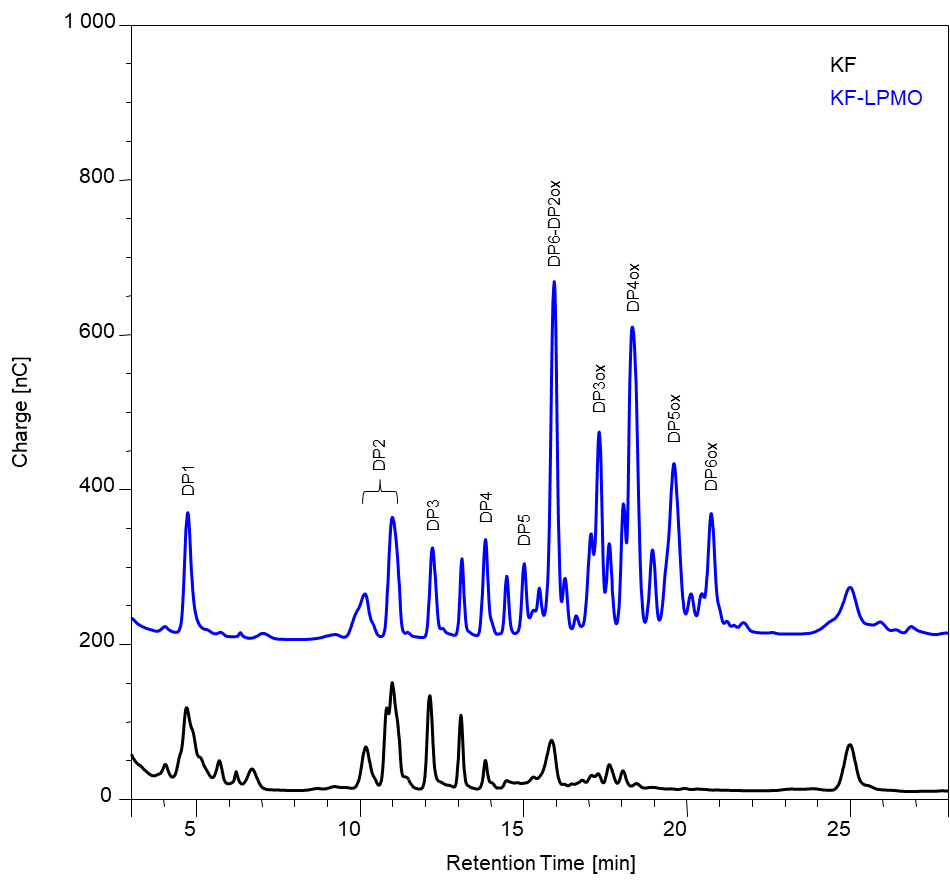

**Fig S3** **a** HPAEC chromatograms showing the elution profile of oxidized and non-oxidized oligosaccharides and **b** quantification by HPAEC analysis of the soluble sugars released by the action of *Pa*LPMO9E on starting bleached birchwood Kraft fibers (KF), *Pa*LPMO9E-treated bleached birchwood Kraft fibers (KF-LPMO), Ultra Turrax dispersed *Pa*LPMO9E-treated fibers (NFC0) and *Pa*LPMO9E-treated fibers submitted to mechanical shearing (NFC1-3). The enzymatic treatment was assessed by four repetition trials that displayed identical elution traces

***LPMO cleaves the cellulose chains***


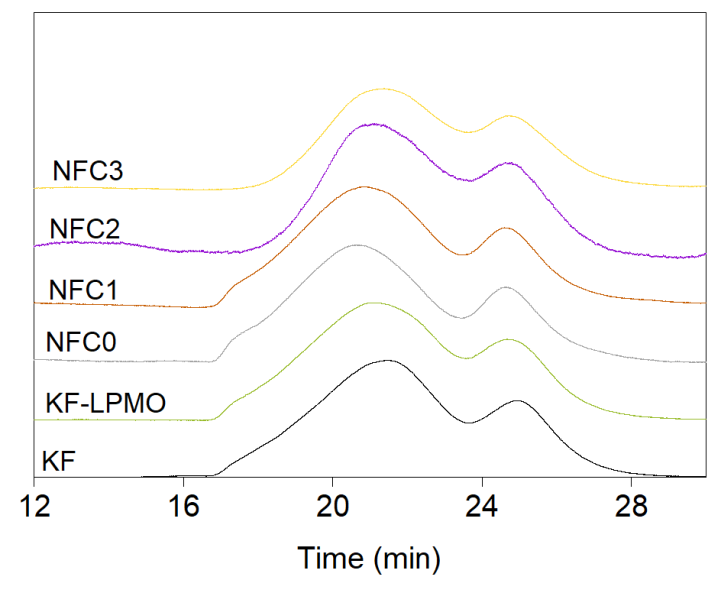


**Fig S4** Normalized HPSEC-RI chromatograms of solubilized fibers from starting bleached birchwood Kraft fibers (KF), *Pa*LPMO9E-treated bleached birchwood Kraft fibers (KF-LPMO), Ultra Turrax dispersed *Pa*LPMO9E-treated fibers (NFC0) and *Pa*LPMO9E-treated fibers submitted to mechanical shearing (NFC1-3)

***LPMO triggers elementary fibrils separation but do not decrease the nanofibers crystallinity***

**Table S1.** Characteristics (chemical shift position of the peaks, δ; and full width at half height, FWHH) of signals generated from the deconvolution at the C-4 region of the solid state ^13^C CP/MAS NMR spectra of starting bleached birchwood Kraft fibers (KF), *Pa*LPMO9E-treated bleached birchwood Kraft fibers (KF-LPMO), Ultra Turrax dispersed *Pa*LPMO9E-treated fibers (NFC0) and *Pa*LPMO9E-treated fibers submitted to mechanical shearing (NFC1-3). Cr, Crystalline; PCr, para-crystalline; AS, accessible surfaces; IAS, inaccessible surfaces; HC, hemicelluloses. Results are expressed as mean ± standard deviation.

| **Assignment** | **Cellulose parts** | **Notation** | **δ (ppm)**  **average** | **FWHH**  **(ppm)** | **Line shape** |
| --- | --- | --- | --- | --- | --- |
| **Cellulose Iα** | Crystalline part | **Cr(Iα)** | 89.43±0.02 | 0.65±0.08 | Lorentzian |
| **Cellulose Iα + Iβ** | Crystalline part | **Cr(Iβ+Iα)** | 88.66±0.01 | 0.80±0.11 | Lorentzian |
| **Paracrystalline cellulose** | Paracrystalline part | **PCr** | 88.47±0.04 | 1.92±0.09 | Gaussian |
| **Cellulose Iβ** | Crystalline part | **Cr(Iβ)** | 87.57±0.04 | 1.06±0.13 | Lorentzian |
| **Cellulose accessible surfaces** | Amorphous part | **AS** | 84.12±0.07 | 1.36±0.16 | Gaussian |
| **Cellulose inaccessible surfaces** | Amorphous part | **IAS** | 83.44±0.21 | 6.24±0.71 | Gaussian |
| **Cellulose accessible surfaces** | Amorphous part | **AS** | 83.07±0.04 | 0.87±0.13 | Gaussian |
| **HC (Xylan)** | Amorphous part | **HC** | 81.74±0.05 | 2.09±0.14 | Gaussian |
